# Rapid, Validated UPLC-MS/MS Method for Determination of Glibenclamide in Rat Plasma

**DOI:** 10.1155/2018/2569027

**Published:** 2018-09-02

**Authors:** Mohd Aftab Alam, Fahad Ibrahim Al-Jenoobi, Abdullah Mohammed Al-Mohizea

**Affiliations:** Department of Pharmaceutics, College of Pharmacy, King Saud University, Riyadh, Saudi Arabia

## Abstract

Quick and specific bioanalytical methods are required for analyzing drugs in biological samples. A simple, quick, sensitive, and specific UPLC-MS/MS method was developed and validated for glibenclamide determination in plasma samples. The plasma samples were processed by protein precipitation technique. Glimepiride was used as internal standard (IS). Glibenclamide and glimepiride were eluted on C18 column (Acquity UPLC®BEH). Mobile phase consisting of acetonitrile (0.1% formic acid) and water (0.1% formic acid) was pumped in binary gradient mode at flow rate of 150 *μ*L/min. Glibenclamide and IS elution time was about 1.0 min, and total run time was 2.0 min. The mass spectrometer (triple-quadrupole) was operated in positive electrospray ionization mode. Sodium adducts [M + Na]^+^ of glibenclamide and IS were monitored in MRM mode. A linear calibration curve was obtained in the range of 10-1280 ng/mL, with regression equation Y = 0.0076 X – 0.0165 and linear regression coefficient r^2^ = 0.999. Lower limit of quantitation was 10 ng/mL. Accuracy of the method at LQC, MQC, and HQC was 109.7% (± 6.7), 93.6% (± 0.4), and 99.3% (± 1.9), respectively. The coefficient of variation for precision at all QC concentrations was less than 6%. Recovery at LLQC, MQC, and HQC was 104.2% (± 4.9), 100.6% (± 0.9), and 102.9% (± 5.8), respectively. The method was successfully implemented for pharmacokinetic investigations (in-house data).

## 1. Introduction

Glibenclamide (glyburide) is a hypoglycemic agent of class sulphonylureas. Glibenclamide (Glynase® PresTab®, micronized tablet) is indicated as an adjunct to diet and exercise to improve glycemic control in adults with type 2 diabetes mellitus (http://www.accessdata.fda.gov/drugsatfda_docs/label/2015/020051s021lbl.pdf). Determination of glibenclamide is required for different purposes including pharmacokinetics, bioequivalence, bioavailability, drug-drug interaction, toxicology, formulation research, and development activities and for forensic purposes. The high pressure liquid chromatography in tandem with ultraviolet, or fluorescence, or diode array, or mass spectrometer detector has been the method of choice.

Khatri et al. developed a fluorescence based HPLC method for determination of glyburide in human plasma samples [1]. Hoizey et al. published a LC-Ion-Trap tandem mass spectrometry method for determination of eight sulfonylureas in blood samples. The samples were prepared by liquid-liquid extraction method with a recovery of < 88%. The method showed good sensitivity, and the glibenclamide elution time was about 5.3 min [2]. Venkatesh et al. estimated six antidiabetic drugs including glibenclamide. The drugs from plasma samples were extracted using liquid-liquid extraction procedure. Mobile phase consisting of 0.01 M formic acid (pH 3.0), acetonitrile, Milli Q water, and methanol was pumped in ternary gradient. The drug elution time was prolonged. Retention time of glibenclamide was 20.7 min [3].

In two separate investigations, the glibenclamide in biological samples was determined using atmospheric pressure chemical ionization in ion trap mass analyzer. Both of these investigations were performed by the same team but under different chromatographic parameters [4, 5]. Villain et al. reported a highly sensitive LC-MS/MS method for determination of glibenclamide in drug facilitated case. Glibenclamide was estimated in hair sample at the concentration range of 5-1000 pg/mg [6]. The analyte was eluted using gradient mode, and Quattro Micro triple-quadrupole mass spectrometer fitted with a Z-Spray ion interface was used for analyses [6]. An LC–MS-MS method was developed to detect the abuse of antidiabetic drugs in racehorses. The method can perform simultaneous detection of 10 antidiabetics in equine plasma and urine [7]. Binz et al. reported a simultaneous identification and quantification method for 5 oral antidiabetics including glibenclamide in serum and hair samples. The LC system was coupled with Waters Xevo TQ MS mass spectrometer [8].

A novel analytical approach for in-line pre-separation and quantification of glibenclamide in tea was developed. A miniaturized and portable automatic multipumping flow system was used to assist the separation and elution of glibenclamide. The eluted drug was determined by fluorometry (*λ*_ex_ = 300 nm; *λ*_em_ = 404 nm) [9]. Li et al. developed an UPLC-MS/MS method for quantification of glibenclamide and puerarin in rat plasma and applied it to investigate pharmacokinetic interaction. The mass spectrometer was operated in positive electrospray ionization (ESI^+^) mode and the protonated molecule [M + H]^+^ was monitored in multiple reaction mode (MRM) [10]. Mistri et al. validated a LC-MS/MS method for estimation of metformin and glibenclamide in human plasma. The acidic acetonitrile was used for protein precipitation of the plasma samples. The drugs were quantified by using a triple-quadrupole mass spectrometer (TQD). TQD was operated in ESI^+^ mode and the protonated molecules [M+H]^+^ were monitored in MRM mode [11]. Contreras et al. evaluated laser-induced breakdown spectroscopy analytic technique for rapid screening and quality control of metformin and glibenclamide. The technique was compared with chromatographic method [12]. Several other analytical methods are also available for determination of glibenclamide or its metabolites in biological fluids [13–15]. Doomkaew et al. developed and validated a fast capillary zone electrophoretic method with photodiode array detection for estimation of metformin, glibenclamide, and gliclazide in raw material and combined tablets [16].

Many of the reported methods have used liquid-liquid extraction and solid phase extraction methods for sample preparation. The liquid-liquid extraction and solid phase extraction methods involve multiple sample processing steps and so they are time consuming.

Aim of present investigation was to develop a simple, quick, sensitive, and specific analytical method for estimation of glibenclamide in plasma samples.

## 2. Materials and Methods

### 2.1. Instrumentation and Reagents

Glibenclamide was purchased from Alfa Aesar (Ward Hill, MA). Glimepiride CRS was obtained as gift sample from SFDA (European Pharmacopeia Standard). HPLC grade methanol (Panreac) was purchased from Panreac Quimica (made in EU). Acetonitrile (HPLC gradient grade) PAI-ACS was purchased from Panreac Quimica. Formic acid was purchased from Loba Chemie Pvt. Ltd. (Mumbai, India). Ultrapure water was prepared by using Milli-QR Gradient A10R (Millipore, Molsheim Cedex, France). The glibenclamide and glimepiride were analyzed using a Waters® Acquity H-Class UPLC®-tandem triple-quadrupole mass spectrometer (TQD) (Waters, Milford, USA). The H-Class UPLC® system comprises Acquity sample manager and Acquity quaternary solvent manager. TQD was equipped with electrospray ionization (ESI) probe. System was controlled by MassLynx 4.1 Software. Data acquisition, processing, and reporting were carried out automatically by using application manager ‘QuanLynx' included with MassLynx 4.1 Software (Version 4.1, SCN 714). The mass tuning was assisted with IntelliStart®. Other instruments included rotary pump (Sogevac, France) for assisting vacuum and a nitrogen generator (Peak Scientific, Scotland) to supply desolvation gas. Argon gas of 99.999% purity was obtained from a local supplier.

### 2.2. Methods

#### 2.2.1. Chromatographic Conditions

Glibenclamide and glimepiride were eluted on an Acquity UPLC®BEH C18 1.7 *μ*m, 2.1 x 50 mm column (Made in Ireland, mfg. part no. 186002350). Analytical column was supported with Acquity UPLC®BEH 1.7 *μ*m VanGuard™ Pre-column 2.1 x 5 mm (Made in Ireland, mfg. part no. 186003975). The column was maintained at 40 ± 5°C by using column heater. The mobile phase consisted of component (A) acetonitrile (0.1 % formic acid) and component (B) water (0.1% formic acid). Mobile phase was pumped at 150 *μ*l/min in gradient mode. The gradient scheme is presented in Table 1. Composition of purge solvent and sample manager wash was acetonitrile (85%) and water (15%). Total sample run time was 2.0 min. The 10 *μ*l sample was injected and the temperature of autosampler was kept at 20 ± 3°C.

#### 2.2.2. Mass Spectrometer Parameters

The glibenclamide (C_23_H_28_ClN_3_O_5_S) and glimepiride (C_24_H_34_N_4_O_5_S, internal standard) were determined using TQD mass spectrometer. TQD was operated in positive electrospray ionization (ESI^+^) mode. Preliminary tuning for glibenclamide and glimepiride was performed through IntelliStart®. The tuning parameters obtained through IntelliStart® were optimized manually in combined mode (LC and fluidics) to improve peak parameters such as selectivity and signal intensity. The cone voltage for glibenclamide and glimepiride was set as 46 (V). Capillary voltage, extractor voltage, and RF lens were set at 2.3 (kV), 3.0 (V), and 0.1 (V), respectively. The source temperature and desolvation temperature were set at 150°C and 250°C, respectively. Nitrogen was used as desolvation gas. Rate of desolvation gas flow was 600 L/H. The flow of cone gas was kept at 0.0 mL. Collision gas (Argon) was flowed at 0.11 mL/min. Low mass resolution (LMR1) and high mass resolution (HMR1) were set as 7.9 and 15.2, while the low mass resolution (LMR_2_) and high mass resolution (HMR_2_) for MS/MS were 10 and 15, respectively. The ion energy (IE_1_) was 0.3. The IE_2_ and gain were set at 1.0. TQD was operated in multiple reaction monitoring mode for determination of glibenclamide (sodium adduct) daughter fragments (m/z 516.1 > 391 and m/z 516.1 > 417) and glimepiride (sodium adduct) daughter fragments (m/z 513.19 > 374.1 and 513.1 > 400). The collision energy (CE) for m/z 516.1 > 391 and m/z 516.1 > 417 fragments was 24 and 20, respectively. The collision energy (CE) for m/z 513.19 > 374.1 and 513.1 > 400 fragments was 24, the same for both fragments. The values for entrance and exit were 2 and 0.2, respectively.

#### 2.2.3. Calibration Curve and Sample Preparation

Standard stock solutions of glibenclamide and glimepiride (IS) were prepared in methanol. The concentration of standard stock solutions was 500 *μ*g/mL. Standard stock solutions were stored in freezer. Series of serially diluted standard solutions (working solutions) of glibenclamide was prepared in methanol-water (80:20) solvent system. These diluted standard solutions were used for preparing the calibration standards in rat plasma. The 100 *μ*L rat plasma was transferred into several Eppendorf tubes. Twenty-microliter (20 *μ*L) aliquot of corresponding diluted standard solution was added to 100 *μ*L blank rat plasma. Ten microliters (10 *μ*L) of diluted internal standard (glimepiride) was added to each plasma sample. The samples were vortexed for 10 sec and then the protein was precipitated by adding 400 *μ*L acetonitrile. Protein precipitated samples were vortexed again for about 20 sec, to uniformly mix the samples. The samples were left over for five minutes and then centrifuged at 10,000 rpm for 6 minutes. The supernatant was separated and analyzed. Five replicates of calibration curve were prepared in the range of 10 to 1280 ng/mL plasma.

## 3. Method Validation

The method was validated by adopting US FDA guidance for bioanalytical method validation (https://www.fda.gov/downloads/Drugs/GuidanceComplianceRegulatoryInformation/Guidances/UCM070107.pdf) [17]. The method was validated for accuracy, precision, linearity, selectivity, recovery, and stability parameters. Three different concentrations of quality control (QC) samples were prepared as LQC (20 ng/mL), MQC (160 ng/mL), and HQC (1280 ng/mL). Along with QC concentrations, the lower limit of quantitation (LLOQ 10 ng/mL) was also used for determination of accuracy and precision.

### 3.1. Accuracy

The accuracy of method describes the closeness of mean test results obtained by the developed analytical method to the true value of the analyte. Accuracy was determined by replicate analysis of processed biological samples containing known amounts of the analyte. For accuracy determination, the blank plasma was spiked with known amount of analyte. Three replicates of LLOQ and of each QC concentration, LQC, MQC, and HQC, were spiked. For accuracy acceptance, the mean of concentration values at LQC, MQC, and HQC should be within 15% of the actual value, while at LLOQ it should not deviate by more than 20% of the actual value. The deviation of the mean from the true value serves as the measure of accuracy [17].

### 3.2. Precision

Precision of biological method is determined by repeated analysis of homogenous samples at different QC concentrations. For present method, the precision was determined at LLOQ and three QC concentrations (LQC, MQC, and HQC). At each QC concentration five replicates were determined. For precision determination, the coefficient of variation (%CV) at LQC, MQC, and HQC levels should not exceed 15%, while at LLOQ the %CV should not exceed 20% [17].

### 3.3. Linearity of Calibration Curve

Five series of eight nonzero serially diluted calibration standards were prepared in 100 *μ*l blank rat plasma. The independent analytical run was performed for each calibration series. The mean of peak area ratio (drug: IS) of all five analytical series was calculated at the corresponding standard concentration and the calibration curve was plotted between standard concentrations versus mean peak area ratio. The linear regression coefficient (r^2^) of calibration curve was calculated. To express the linearity of the analytical method, the linear regression coefficient (r2) of calibration curve in the concentration range of 10-1280 ng/mL was calculated by linear regression analysis. For calibration curve, 20% deviation at lower limit of quantitation, but 15% deviation at other calibration standards from nominal concentration, was accepted. At least four out of eight nonzero standards should meet this criteria, including lower limit of quantitation and upper limit of quantitation [17].

### 3.4. Lower Limit of Quantitation (LLOQ)

Lowest standard concentration on the calibration curve was considered as the LLOQ. LLOQ was determined at least at a five-time higher analyte response than the blank noise (baseline) at the retention time of the analyte. The criterion for LLOQ was as follows: at LLOQ the analyte peak (response) should be identifiable, discrete, and reproducible with a precision of 20% CV and accuracy of 80-120% [17].

### 3.5. Selectivity

Blank plasma samples from five rats were collected and analyzed separately to observe any interactive chromatogram at analyte retention time. Response of blank plasma samples was compared with the blank plasma samples spiked at LLOQ (10 ng/mL). The absence of any interactive chromatogram at the retention time of the analyte indicates selectivity of the method [17]. The accuracy of the method at LLOQ should not deviate by more than 20%.

### 3.6. Recovery (Matrix Effect)

Recovery of a bioanalytical analytical method is related to the drug extraction efficiency of the method within the limits of variability. Recovery of analyte in the plasma sample was determined at LLOQ, MQC, and HQC concentrations. The signal response (area) of the QC concentrations was determined in plasma samples and compared with the signal response of aqueous samples (samples of the same concentrations prepared by the same procedure in 100 *μ*l water in place of plasma). Percent recovery was calculated as follows: dividing the peak area of analyte extracted from plasma/peak area of analyte in aqueous samples X 100 [17].

### 3.7. Post-Preparative Stability

The Post-preparative stability study was carried out using samples of LLOQ, LQC, MQC, and HQC. For post-preparative stability determination, the supernatants of processed samples were stored at laboratory temperature (23 ± 1°C) for 24 hours. Analyte concentrations of post-preparative stability samples were determined and compared with freshly prepared analytical results.

### 3.8. Carryover

To determine the carryover effect of the method, the blank plasma samples were analyzed just after the analysis of the upper limit of quantitation sample. The carryover was accepted if the blank sample response at analyte retention time was not more than 20% of the mean response of LLOQ.

## 4. Results

**Figure 1:**
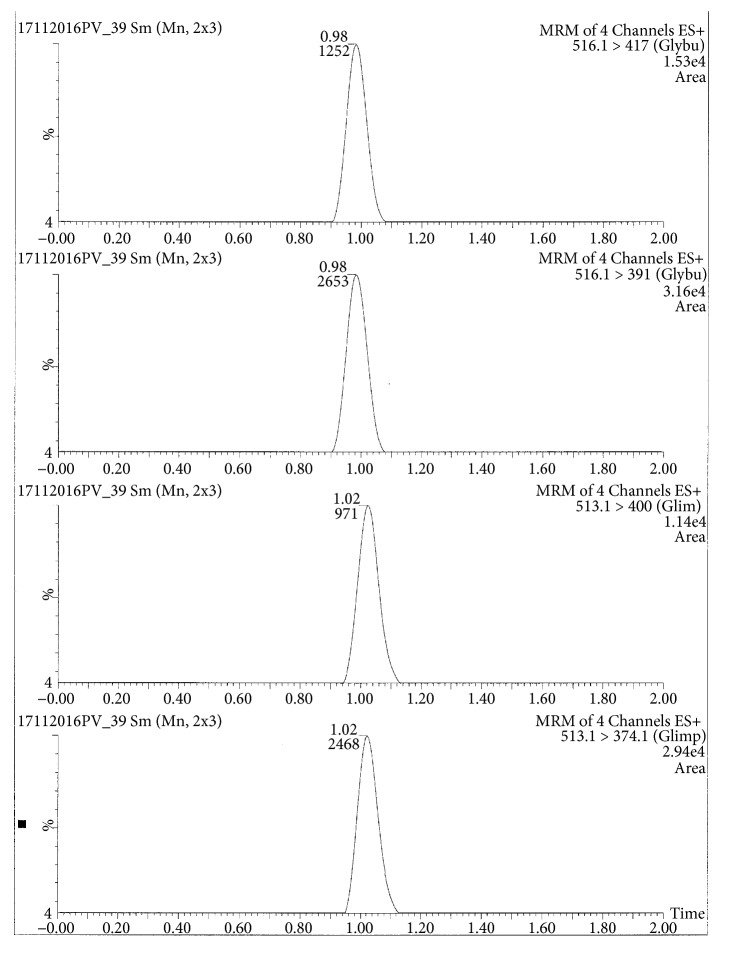
The representative chromatograms of daughter fragments of glibenclamide and glimepiride.

**Figure 2:**
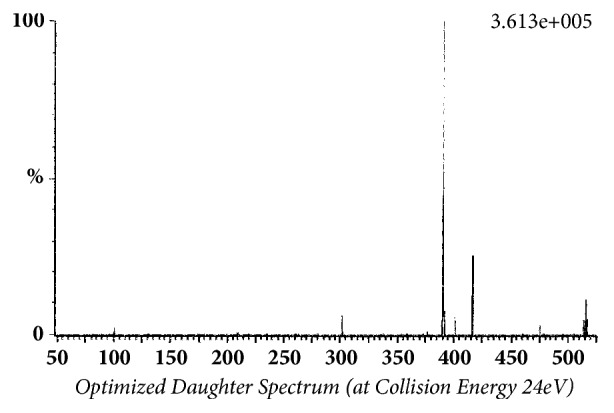
Transition of glibenclamide sodium adduct to daughter fragment (m/z 516.11 > 391).

**Figure 3:**
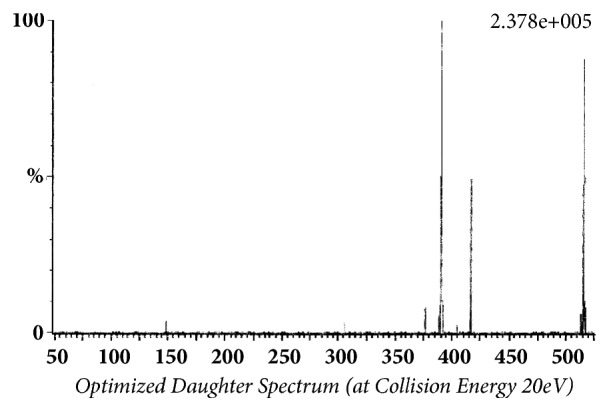
Transition of glibenclamide sodium adduct to daughter fragment (m/z 516.11 > 417).

**Figure 4:**
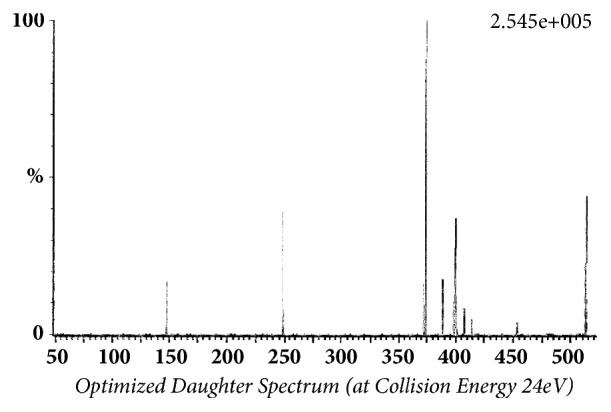
Transition of glimepiride sodium adduct to daughter fragment (m/z 513.19 > 374).

**Figure 5:**
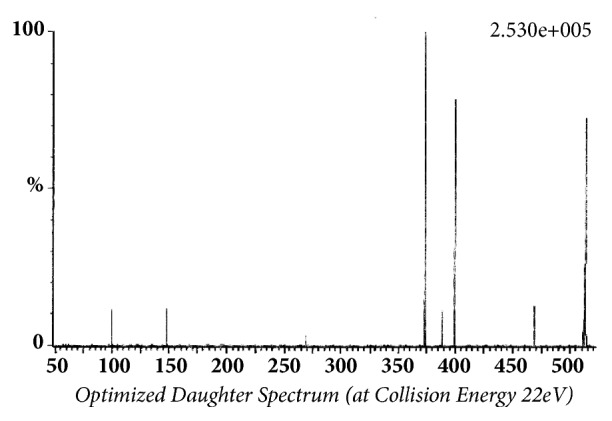
Transition of glimepiride sodium adduct to daughter fragment (m/z 513.19 > 400).

Glibenclamide and glimepiride were eluted on Acquity UPLC®BEH C18 (1.7 *μ*m, 2.1 x 50 mm) column in gradient mode using acetonitrile (0.1% formic acid) and water (0.1% formic acid) as mobile phase. The analyte elution time was short, 0.98 min for glyburide and 1.02 min for glimepiride. The representative chromatograms of analyte fragments are presented in Figure 1. Mass detector (TQD) was tuned in positive electrospray ionization (ESI^+^) mode, and the sodium ion adducts of glibenclamide [M + Na]^+^ and glimepiride [M + Na]^+^ were monitored in multireaction monitoring (MRM) mode. The parent sodium ion [Na^+^] adduct of glibenclamide was observed at m/z 516.11. The parent sodium ion [Na^+^] adduct of glimepiride was observed at m/z 513.19. Parent sodium ion adducts of glibenclamide and glimepiride were fragmented into daughter fragments, in multiple reaction monitoring mode. The molecular masses of daughter fragments of glibenclamide sodium ion adduct (m/z 516.11) were 391 and 417; the molecular masses of daughter fragments of glimepiride sodium ion adduct (m/z 513.19) were 374 and 400. Optimized daughter spectrums of glibenclamide fragments are presented in Figure 2 (m/z 516.11 > 391) and Figure 3 (m/z 516.11 > 417). Optimized daughter spectrums of glimepiride fragments are presented in Figure 4 (m/z 513.19 > 374) and Figure 5 (m/z 513.19 > 400).

**Figure 6:**
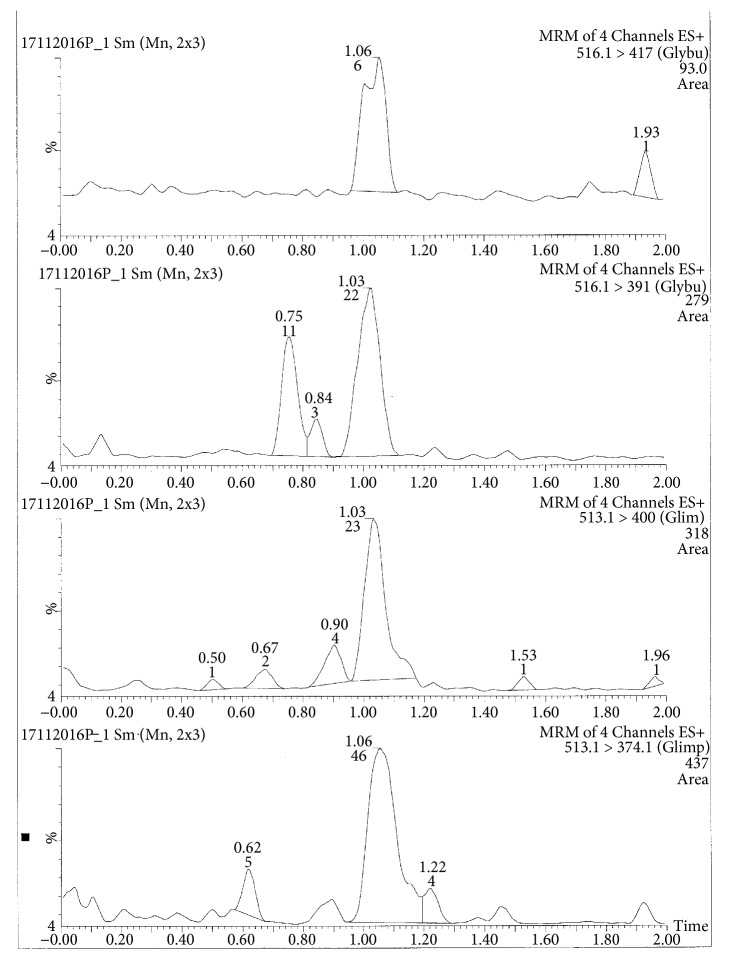
The representative chromatograms of blank plasma sample.

**Figure 7:**
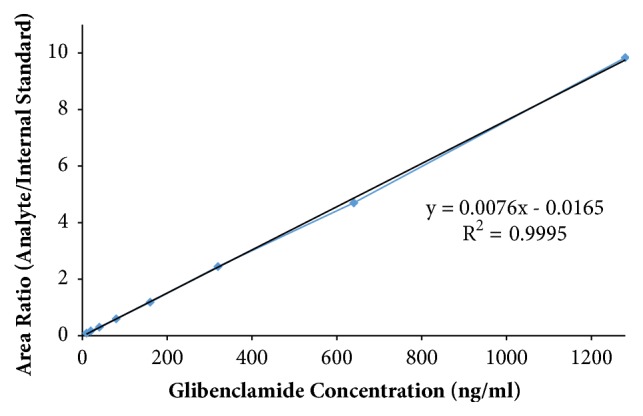
The calibration curve of glibenclamide in plasma.

The mean area of noise signal at analyte retention time from five different blank plasma samples obtained from five different rats was 13.6 ± 4.7. The ratio of signal response (area) produced by blank sample (noise peak) with signal response (area) at LLOQ was 1: 10.8. The LLOQ and LQC for glibenclamide were determined as 10 ng/mL and 20 ng/mL, respectively. In blank plasma samples, there was no major interfering peak at the retention time of glibenclamide, so the method is considered selective, Figure 6. Linear regression equation for calibration curve was calculated as Y = 0.0076 X – 0.0165, Figure 7. The linear regression coefficient (r^2^) for the calibration curve was 0.999. Validation results are presented in Table 2. The accuracy of the method at LLOQ, LQC, MQC, and HQC was 117% (± 3.8), 109.7% (± 6.7), 93.6% (± 0.4), and 99.3% (± 1.9), respectively. The coefficient of variation at all QC concentrations was less than 6%. The values of %CV within suggested limits indicate good precision of the method. The recovery at LLOQ, MQC, and HQC was 104.2% (± 4.9), 100.6% (± 0.9), and 102.9% (± 5.8), respectively. For stability study, after 24 hours storage, the glibenclamide content at LLOQ and at QC concentrations LQC, MQC, and HQC was estimated as 9.9 ± 0.6 ng/mL, 20.3 ± 1.3 ng/mL, 152.5 ± 8.7 ng/mL, and 1234 ± 33.0 ng/mL, respectively. No significant carryover was estimated in blank plasma samples run after upper limit of quantitation sample.

## 5. Discussion

An UPLC-MS/MS method for estimation of glibenclamide in plasma samples was developed and validated. The mobile phase flowing in gradient mode provides sharp and symmetric peaks of analyte and internal standard. No significant tailing was noticed in chromatograms. In mass tuning, the method has different observation from prior art publications. While tuning, protonated glibenclamide and glimepiride molecules [M + H^+^] were observed [6, 18, 19], but the signal intensity of their peaks were weak. The signal intensity of sodium ion [Na^+^] adducts of glibenclamide as well as of glimepiride was significantly high than their protonated molecules [M + H^+^]. Therefore, the final tuning was performed after selecting sodium ion [Na^+^] adducts of glibenclamide and glimepiride. The molecular mass of highest intensity peaks of glibenclamide and glimepiride daughter fragments was 391 and 374, respectively. Therefore, the daughter fragments having a molecular mass of 391 and 374 were selected as quantifying ions for glibenclamide and glimepiride, respectively. Molecular mass of qualifying ions of glibenclamide and glimepiride was 417 and 400, respectively.

For developing LC-MS/MS methods, the most preferred internal standard is deuterated isotope of the analyte in question. If deuterated isotope of the analyte is not available, then the next choice for internal standard is the molecule of close molecular weight and almost similar molecular structure, that of analyte. The constant recovery by a common sample preparation procedure is another attribute required for the internal standard molecule. The closeness of molecular weight and the similarity in chemical structure and physicochemical properties provide an opportunity to select the common sample preparation procedure and the common operating conditions in mass spectrometer and liquid chromatography. The molecular weight of glibenclamide and glimepiride is 494 and 490.6, respectively. Like molecular weight, their molecular structures also showed similarity (sulfonylurea derivatives). Method of protein precipitation was used for preparing the samples for analysis. Acetonitrile was added to precipitate the protein of plasma samples. The precipitated protein was separated by centrifuging the samples. Clear supernatant was removed for the analysis. The recovery through single step protein precipitation method was good and repeatable for glimepiride (internal standard) and glibenclamide (see recovery results). Protein precipitation is a short and simple process as compared to other methods of sample preparation. The mass tuning parameters (cone voltage and collision energy) were close for glibenclamide as well as for glimepiride. The constant/repeatable recovery and close mass tuning parameter suggested that glimepiride is suitable internal standard.

The operation of TQD (MS/MS) in multireaction monitoring mode provides higher selectivity. There was no interference of endogenous components of plasma, in blank plasma sample. Calibration was linear as determined over eight serially diluted concentrations and five replicates at each level. Lower limit of quantitation and limit of quantitation were determined based on signal to noise ratio. LLOQ was taken as at least five-time area to the area of blank sample (noise ratio) at the retention time of the analyte and reproducible response with a maximum of 20% bias. LQC was taken as double of LLOQ. For accuracy determination, the plasma samples were spiked with known concentrations of glibenclamide. Results of accuracy investigations showed that the analyzed concentrations were close to the true value of the glibenclamide in the respective sample. The outcome of accuracy study suggested that developed method accurately analyzes the glibenclamide in plasma within the proposed linearity range of calibration curve. For precision investigation, the blank plasma was spiked with QC concentrations of glibenclamide. Recovery of glibenclamide was calculated against spiked aqueous solutions prepared in the same way as plasma samples. The extent of recovery of glibenclamide and of the glimepiride was consistent and reproducible. The spiked plasma samples of QC concentrations (aspirated samples packed in airtight tube) were stable over at least 24 hrs at laboratory temperature (24 ± 2°C).

## 6. Conclusion

The developed method provides an alternate approach for the estimation of glibenclamide. Since the signal intensity of the sodium ion adduct of glibenclamide was better than the signal intensity of protonated molecular ion. Method was successfully validated for glibenclamide estimation in multiple reaction monitoring mode. The method is simple, fast, accurate, and precise. It was successfully applied for pharmacokinetics investigations of glibenclamide.

## Figures and Tables

**Table 1:** Representing gradient scheme of mobile phase.

**Time in min**	**Mobile phase flow**	**Mobile Phase**	
		**Component A**	**Component B**
0.0	150 *μ*l/min	85	15

0.70	150 *μ*l/min	85	15

0.71	150 *μ*l/min	100	0

0.80	150 *μ*l/min	100	0

0.81	150 *μ*l/min	85	15

2.0	150 *μ*l/min	85	15

**Table 2:** Results of validation studies.

**Validation Parameter**	**Validation Results**			
	**LLOQ**	**LOQ**	**MLOQ**	**HLOQ**
**QC Samples**	10 ng/mL	20 ng/mL	160 ng/mL	1280 ng/mL

**Accuracy**	117 ± 3.8 %	109.7 ± 6.7 %	93.6 ± 0.4 %	99.3 ± 1.9 %

**Precision **	4.52 %CV	5.94 %CV	1.55 %CV	2.08 %CV

**Linearity**	10 – 1280 ng/mL			

**Recovery**	104.2 ± 4.9 %	-* *-* *-* *-* *-* *-* *-* *-* *-* *-* *-* *-* *-* *-* *-* *-* *-	100.6 ± 0.9 %	102.9 ± 5.8 %

**Stability**	9.9 ± 0.6 ng/mL	20.3 ± 1.3 ng/mL	152.5 ± 8.7 ng/mL	1234 ± 33.0 ng/mL

## Data Availability

The data used to support the findings of this study are included within the article.
