# Erratum to “Rapid, Validated UPLC-MS/MS Method for Determination of Glibenclamide in Rat Plasma”

**DOI:** 10.1155/2019/6470528

**Published:** 2019-02-03

**Authors:** Mohd Aftab Alam, Fahad Ibrahim Al-Jenoobi, Abdullah Mohammed Al-Mohizea

**Affiliations:** Department of Pharmaceutics, College of Pharmacy, King Saud University, Riyadh, Saudi Arabia

In the article titled “Rapid, Validated UPLC-MS/MS Method for Determination of Glibenclamide in Rat Plasma” [1], an acknowledgment should be added as follows:

“Authors thank the Research Center, College of Pharmacy, and Deanship of Scientific Research, King Saud University for funding this research project.”
